# Ternary copper(II) complex: NCI60 screening, toxicity studies, and evaluation of efficacy in xenograft models of nasopharyngeal carcinoma

**DOI:** 10.1371/journal.pone.0191295

**Published:** 2018-01-12

**Authors:** Munirah Ahmad, Shazlan-Noor Suhaimi, Tai-Lin Chu, Norazlin Abdul Aziz, Noor-Kaslina Mohd Kornain, D. S. Samiulla, Kwok-Wai Lo, Chew-Hee Ng, Alan Soo-Beng Khoo

**Affiliations:** 1 Molecular Pathology Unit, Cancer Research Centre, Institute for Medical Research, Kuala Lumpur, Malaysia; 2 Department of Pathology, Faculty of Medicine, Universiti Teknologi MARA, Sungai Buloh, Selangor, Malaysia; 3 Aurigene Discovery Technologies Limited, Bangalore, India; 4 Department of Anatomical & Cellular Pathology, State Key Laboratory in Oncology in South China and Li Ka Shing Institute of Health Science, The Chinese University of Hong Kong, Hong Kong, China; 5 Department of Pharmaceutical Chemistry, School of Pharmacy, International Medical University, Kuala Lumpur, Malaysia; 6 Institute for Research, Development and Innovation, International Medical University, Kuala Lumpur, Malaysia; Duke University School of Medicine, UNITED STATES

## Abstract

Copper(II) ternary complex, [Cu(phen)(C-dmg)(H_2_O)]NO_3_ was evaluated against a panel of cell lines, tested for *in vivo* efficacy in nasopharyngeal carcinoma xenograft models as well as for toxicity in NOD scid gamma mice. The Cu(II) complex displayed broad spectrum cytotoxicity against multiple cancer types, including lung, colon, central nervous system, melanoma, ovarian, and prostate cancer cell lines in the NCI-60 panel. The Cu(II) complex did not cause significant induction of cytochrome P450 (CYP) 3A and 1A enzymes but moderately inhibited CYP isoforms 1A2, 2C9, 2C19, 2D6, 2B6, 2C8 and 3A4. The complex significantly inhibited tumor growth in nasopharyngeal carcinoma xenograft bearing mice models at doses which were well tolerated without causing significant or permanent toxic side effects. However, higher doses which resulted in better inhibition of tumor growth also resulted in toxicity.

## Introduction

Resistance and toxicity of currently available chemotherapeutic agents remain major problems in cancer therapy. Therefore, new anticancer drugs with greater effectiveness but reduced toxic side effects would be most useful. Fewer platinum compounds are now entering clinical trials and this has been attributed to not only their inherent resistance, systemic toxicity and severe side effects but also due to a shift in the design and development of anticancer metallodrugs [1–5]. Hambley provides a basis of designing such metallodrugs, viz. *via* the function of the metal and ligand moieties [6]. These are (i) the metal complex is active in its inert form, (ii) the metal complex is active in its reactive form, (iii) the metal serves as a radiation enhancer, (iv) the compound contains a radioactive metal, (v) the metal or its biotransformation product is active, (vi) a ligand is biologically active, and (vii) only a fragment of the complex is active [6]. Another approach in designing metallodrugs is based on cancer genomics and this is directed at cell survival pathways, mechanism of resistance, metastasis, and drug-specificity arising from its pre-defined chemical and biological reactivity [7].

There is also a shift to exploring and developing anticancer metal-based drugs involving non-platinum metals like copper [1, 2, 8–10]. Copper complexes have been highlighted to be promising alternatives to platinum-based drugs due to the elevated need for copper by cancer tissues, established role of copper in tumor angiogenesis, and due to an altered metabolism of copper in many types of tumor [11, 12]. There is also increasing acceptance that the mechanism of action of copper complexes is distinctly different, thereby providing the possibility of circumventing the problems encountered by platinum drugs. Additionally, the copper complexes are also reported to have a broader spectrum of activities and lower toxicity, and are able to overcome inherited and/or acquired resistance to cisplatin.

The review by Santini *et al*. found extensive research on copper complexes with anticancer properties reported between the years 2008 and 2012 but more work is needed to establish clear correlation between the *in vitro* antitumor activity of these complexes with the oxidation state, coordination number, or geometry [9]. In spite of this shortcoming, recent anticancer screening of copper(II) complexes still shows promising *in vitro* results [13–15]. However, recent *in vivo* studies of copper compounds are few and the tested compounds are copper(II) schiff base derived from 3-(3-phenyl-allylidene)pentane-2,4-dione, copper(II) thiosemicarbazone, trinuclear CuSn_2_(Trp) complex, copper carbonate-folic conjugated nanoparticles and Casiopeína’s group of copper(II) complexes [16–20]. *In vivo* administration of a copper(II) thiosemicarbazone, Cu(GTSC) significantly inhibited tumor growth in HCT116 (colon cancer cell line) xenografts in nude mice [17].

Nasopharyngeal cancer (NPC) is prevalent in southern China, Southeast Asia and northern Africa [21]. Malaysia has the highest incidence rate of NPC in the world [22]. Among men, NPC is the fifth most common cancer in Malaysia and most patients present at late stage [23, 24]. NPC usually presents at late stage because early stage of NPC may be asymptomatic or present with apparently trivial signs [25]. Radiotherapy and/or concurrent chemo-radiotherapy (CRT) is the treatment modality for NPC. Late toxicities remain a concern in the management of NPC [26, 27].

**Fig 1:**
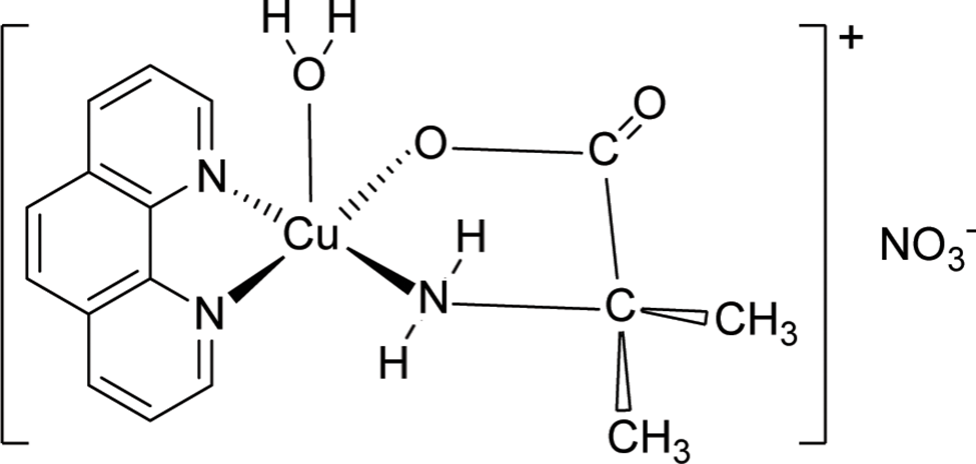
The structure of [Cu(phen)(C-dmg)(H_2_O)]NO_3_ with the Cu(II) complex as a square pyramidal cation and an uncoordinated nitrate anion.

The current study is a continuation of our recent *in vitro* investigation of the anticancer property of a series of ternary copper(II) complexes, [Cu(phen)(aa)(H_2_O)]NO_3_∙xH_2_O (phen = 1,10-phenanthroline; aa = glycine, DL-alanine, sarcosine, α-dimethylglycine) [28, 29]. Here, we studied [Cu(phen)(C-dmg)(H_2_O)]NO_3_ (C-dmg = α-dimethylglycine) (Fig 1) (hereafter called Cu(II) complex), in which the chelated amino acid is non-proteinogenic. *In vivo* efficacy studies were performed using NOD scid gamma female mice (NSG) which were subcutaneously xenografted with nasopharyngeal carcinoma HK1 or C666-1-GFP-Luc2 cells. The mice were also evaluated for toxicity. We found that the Cu(II) complex could inhibit tumor growth at doses which was not associated with toxicity.

## Results and discussion

### NCI60 five-dose screening

The [Cu(phen)(C-dmg)(H_2_O)]NO_3_ (Cu(II) complex) was submitted to the National Cancer Institute (NCI number S774845) for NCI60 human cell line screening. The panel consists of 60 human cancer cell lines from a variety of cancers, viz. leukemia, non-small cell lung (NSCLC), colon, central nervous system (CNS), melanoma, ovarian, renal, prostate and breast cancers. Five dose (0.01 μM -100 μM) antiproliferative and cytotoxicity analysis were done and the data was used to calculate 50% growth inhibition of tested cells (GI50), total growth inhibition (TGI), and lethal dose concentration inducing 50% cell death (LC50; raw values are available in (S1 Fig). Analysis of these data shows that the Cu(II) complex has a mean GI50 and LC50 values of 1.51 and 9.12 μM respectively. Comparison with those of cisplatin (with corresponding values of 1.49 and 44.00 μM; cisplatin NSC number 119875) reveals that this complex has equivalent activity based on 50% growth inhibition but is fivefold more cytotoxic.

**Fig 2:**
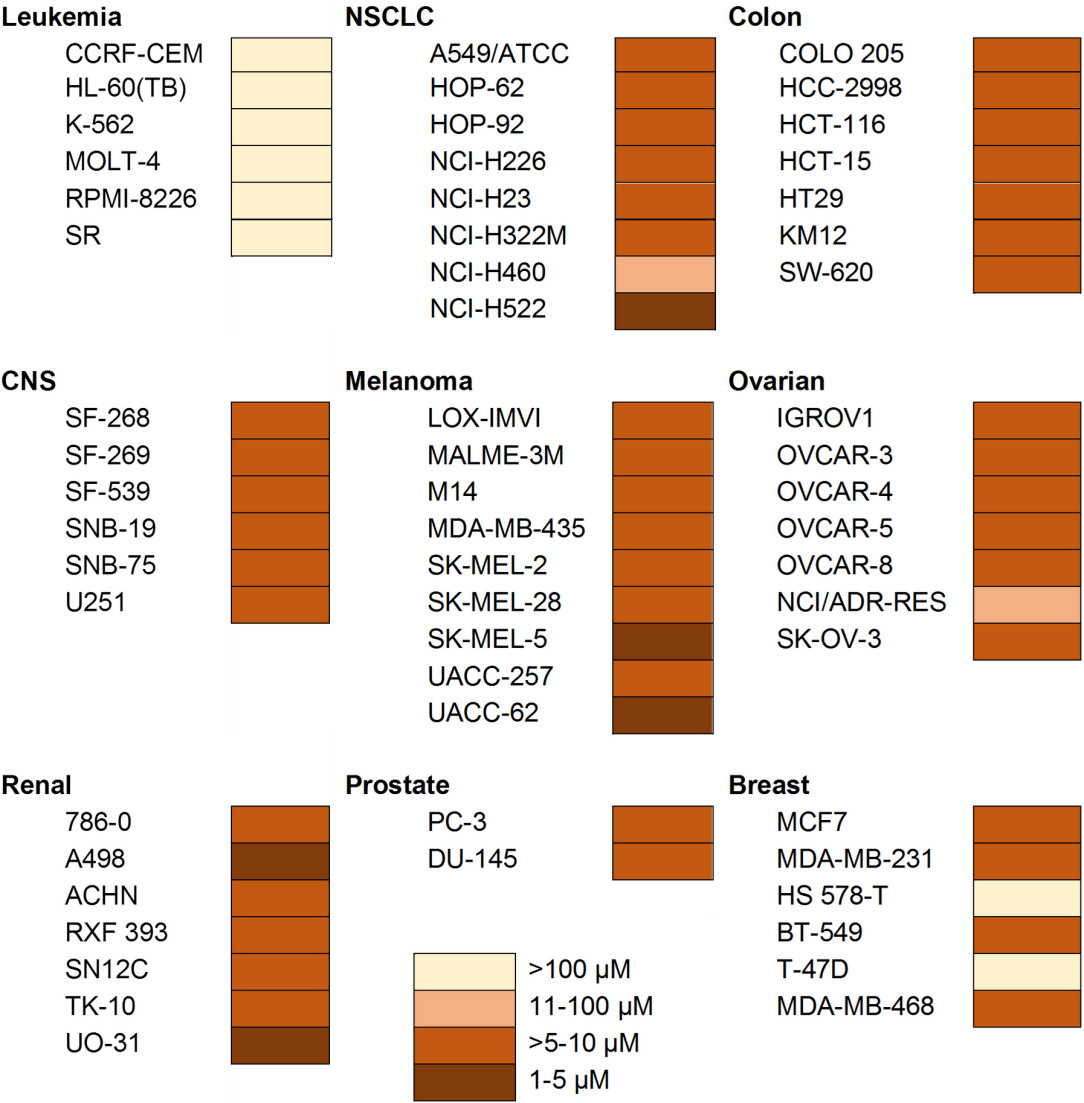
Heat map data representing LC50 concentrations of Cu(phen)(C-dmg)NO_3_ across the NCI-60 human cancer cell line panel. Concentration ranges from most cytotoxic (1–5 μM) to least cytotoxic (>100 μM).

A heat map was constructed to visualize the Cu(II) complex LC50 concentrations for the cell lines in the NCI60 panel (Fig 2). In terms of LC50 values, the Cu(II) complex is cytotoxic (>5–10 μM) or strongly cytotoxic (1–5 μM) [30] towards a broad range of cell lines and most of the panel of cell lines (viz. NSCLC, colon, CNS, melanoma, ovarian, and prostate). This is the most striking feature and consequently, its anticancer effectiveness can be termed as broad spectrum. However, certain cell types appeared more resistant, suggesting that the Cu(II) complexes are not merely displaying general toxicity but have some selectivity. This is in concordance to our previous report in which the Cu(II) complexes were more toxic to cancer cells compared to non-cancer cells [28, 29]. Therefore, the mechanism of cell killing could be specific for certain cellular characteristics. The leukemia panel of cell lines tested (CCRF-CEM, HL-60(TB), K-562, MOLT-2, RPMI-8226, and SR) and breast cancer lines (T47D and HS578T) were resistant to the Cu(II) complex with LC50 values of more than 100 μM.

One of the causes of drug resistance is the overexpression of a family of 49 identified cell membrane transporter proteins which promote chemotherapeutic drug efflux [31]. Although not all have been thoroughly studied, some of these proteins have binding specificity to different substrates while others have overlapping substrate binding specificity. Multiple drug resistance 1 (MDR1; also called Pgp) and BCRP transporter proteins have been identified in leukemias and breast cancer, and they have been correlated or associated with chemoresistance in these cancer cell types respectively [32]. Most of the NCI leukemia cell lines were also reported to be resistant to [Cu(o-phthalate)(phenanthroline)] [30]. Strikingly, all renal cell lines were highly sensitive (LC50 values of 1–10 μM) to the present Cu(II) complex, in contrast to their resistance towards some Iridium(III) organometallic complexes [33]. The chemoresistance towards these Iridium(III) complexes was ascribed to the renal cell lines having a high abundance of multi-drug resistant MDR1 protein (Pgp) expression [32]. Taken together, this suggests that, the mechanism of resistance to copper complexes may be different from the mechanism of resistance to iridium (III) complexes. This also suggests that alteration of the design of metallodrug may be a possible approach to overcome drug resistance [31].

### Cytotoxicity evaluation using rat primary hepatocytes

**Fig 3:**
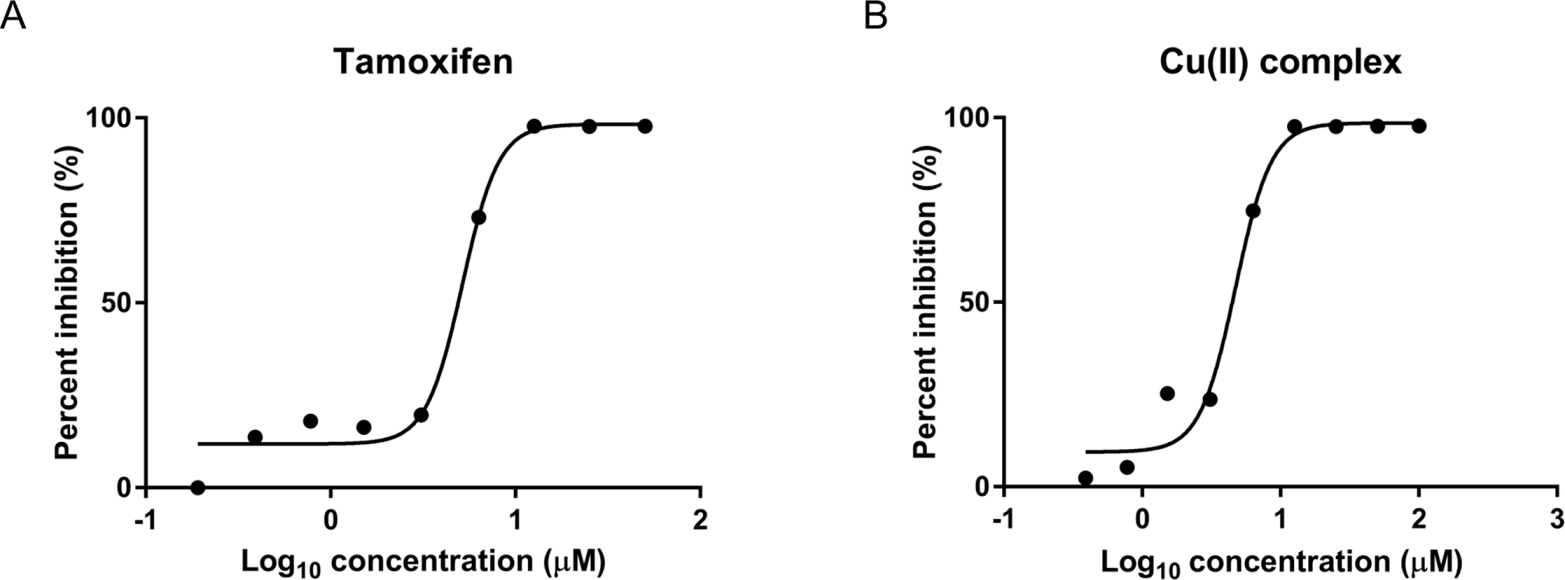
Cytotoxicity profile of tested compounds on rat primary hepatocytes. Dose-response curve of hepatocytes exposed to Tamoxifen as standard (A) and Cu(II) complex (B) for 72 h. The viability was determined by resazurin assay and the IC50 of Cu(II) complex and tamoxifen are 4.7 μM and 5.1 μM respectively.

Drug-induced liver damage, i.e. hepatotoxicity, is a serious side effect of anticancer drugs. Among the *in vitro* models for testing hepatotoxicity, rat primary hepatocytes is one of the two types of cells that are extensively used [34]. Cytotoxicity assessment using rat primary hepatocytes was performed using a standard method which was reported to be a robust method in preclinical safety settings [34, 35]. Briefly, a series of wells, each containing 12.0 x 10^3^ viable hepatocytes in William’s medium, were incubated for 72 h at 37°C with decreasing concentrations of the Cu(II) complex (100, 50, 25, 12.5, 6.25, 3.125, 1.56, 0.78 and 0.39 μM) respectively. The percentage cytotoxicity was calculated as (100—viability) %. The Cu(II) complex exhibited cytotoxicity at concentrations over 3 μM (Fig 3) and the cytotoxic concentration that kills 50% of the cells (IC50) was found to be 4.7 μM. The IC50 of the anticancer drug, Tamoxifen (used as reference standard) was also determined and its value was 5.1 μM.

### Assessment of CYP450 induction/inhibition

Assessment for potential drug-drug interaction is an important component of drug discovery and development [36]. About 25% of adverse drug reactions were caused by drug-drug interactions [36]. Most drug-drug interaction and drug metabolism occur in the hepatic cytochrome P450 (CYP) system [37]. CYP is a superfamily of metabolizing enzymes located primarily in hepatocytes. Inhibition or induction of CYP enzymes is a mechanism for drug-drug interaction. In the current work, the inhibition and induction of CYP enzymes were investigated. CYP induction assay was performed using rat primary hepatocytes for CYP 1A and 3A isoforms with dexamethasone (30 μM) and 3-methyl cholanthrene (1.0 μM) as probe substrates, respectively. Cytochrome CYP 3A enzymes are considered to be the most important enzymes catalyzing drug metabolism with broad substrate specificity while CYP 1A enzymes play key roles in the metabolic activation of aromatic hydrocarbons to carcinogenic metabolites [38–40]. In orally administered anticancer drug, gut wall CYP 3A enzyme is considered important for drug-drug interactions and xenobiotic interactions [37]. The results of the CYP 450 induction assay are tabulated in Table 1. The Cu(II) complex tested showed less than two folds of induction (<13% of positive control) in both tested CYP isoforms (enzymes) when compared to respective basal control (normal/untreated cells). Dexamethasone (potent inducer of rat CYP 3A) [39] and 3-methylcholanthrene (3-MC; potent inducer of CYP 1A) [40], which were used as reference standards (positive controls) showed more than 10 fold induction of both CYP 3A and 1A isoforms in comparison with basal controls (untreated cells), indicating validity of the assay. As the fold induction by the Cu(II) complex was not more than 40% of positive control [41], the results suggested that the Cu(II) complex did not cause significant induction of both CYP 3A and 1A isoforms under the tested conditions. This is favorable to the Cu(II) complex because induction of CYP 3A and CYP 1A is known to metabolize aromatic compounds into carcinogenic metabolites [40]. Nevertheless, further evaluation on human hepatocytes would be useful as there could be species-dependent metabolism [42].

**Table 1:** Induction of CYP enzymes CYP 1A and 3A by standard drugs and Cu(II) complex.

Compounds	Fold induction		% of positive control	
	CYP3A	CYP1A	CYP3A	CYP1A
Dexamethasone (30.0 μM)*	27.3	—	—	—
3-Methyl cholanthrene (1.0 μM)*	—	10.9	—	—
Cu(II) complex (1.0 μM)**	0.8	0.8	3.0	7.4
Cu(II) complex (0.3 μM)	1.2	1.4	4.3	13.0
Cu(II) complex (0.1 μM)	1.3	1.1	4.7	10.1

* Known reference standards (positive controls).

** Cell death observed (approximately 30%).

**Fig 4:**
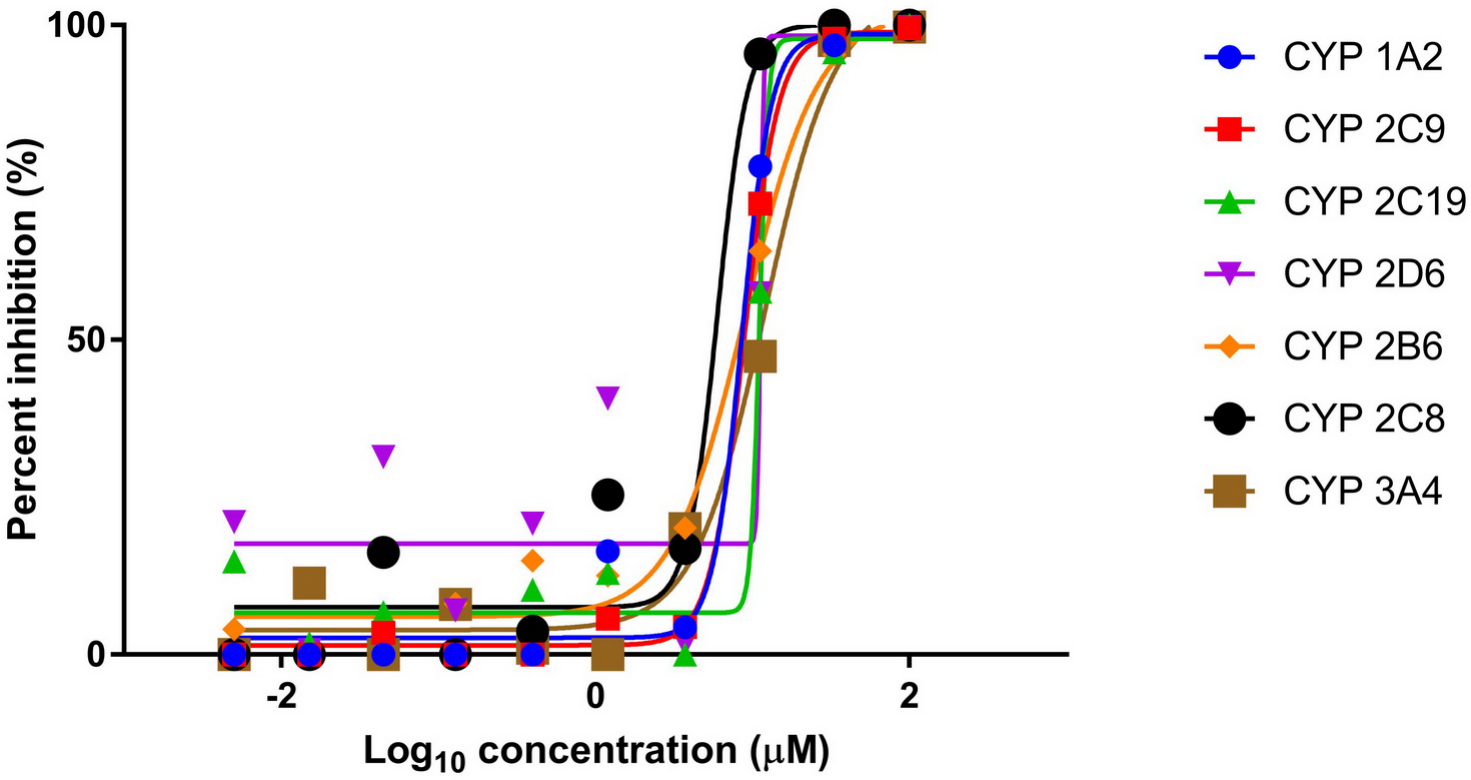
Inhibition of CYP enzymes by Cu(II) complex: Plot of percent inhibition versus concentration of Cu(II) complex. The inhibitory activity of Cu(II) complex was examined on seven CYP isoforms using pooled human liver microsomes and pooled probe substrate. The complex showed IC50 values of between 5 to 10 μM against the tested isoforms.

In the CYP inhibition assay, human liver microsomes were incubated with the Cu(II) complex or reference standard. The inhibitory effects of these compounds were investigated against the major CYP isoforms namely 1A2, 2C9, 2C19, 2D6, 2B6, 2C8 and 3A4 with their respective isoform probe substrates in one reaction mixture (pooled probe substrate format using LC-MS/MS). The percent inhibition curves against all the tested isoforms by the Cu(II) complex were plotted (Fig 4). The IC50 values of the Cu(II) complex were found to be approximately in the range 5–10 μM against all tested isoforms, showing that it may be a moderately potent inhibitor of these tested metabolic CYP enzymes (Table 2). The probe substrates of the reference standards have IC50 values of between 0.05–1.47 μM. This suggests that there could be inhibition of metabolism of drugs and endogenous compounds which are substrates of these CYP enzymes resulting in a potential drug-drug interaction.

**Table 2:** IC50 values of CYP enzyme inhibition by Cu(II) complex and reference standards.

		IC50 (μM)	
CYP Isoform	Cu(II) complex	Reference standards (Drugs)	
1A2	8.01	0.16	(Fluvoxamine)
2C9	8.56	0.16	(Sulfaphenazole)
2C19	10.26	1.47	(Fluoxamine)
2D6	10.23	0.43	(Quinidine)
2B6	7.59	0.10	(Ticlopidine)
2C8	10.32	0.31	(Quercetine)
3A4	5.46	0.05	(Ketoconazole)

### Acute *in vivo* toxicity of Cu(II) complex

Acute toxicity caused by the complex was tested on a method modified from the NCI single dose method [43]. The experiment was performed to find the maximum tolerated dose (MTD) for the calculation of doses for repeat-dose animal testing; the experiment was designed to minimize both the use of compound tested and the number of animals to be sacrificed. The MTD is defined as the maximum dose administered to the animal that does not result in drug-related lethality or body weight loss of equal or greater than 20 percent [44]. Five doses, viz. 35.0, 17.5, 12.5, 7.5 and 5.5 mg/kg body weight of the Cu(II) complex, were injected intraperitoneally as single doses into each mouse respectively and the mice were observed daily for 14 days for drug-induced toxicity. The findings of the acute toxicity study are listed in S1 Table. After 15 minutes of a single dose of 35 mg/kg of Cu(II) complex, the mouse showed signs of toxicity for the first 4 hours, such as immobility as well as unresponsiveness to provocation, and was found dead on the next day. Mice administered with 17.5 and 12.5 mg/kg doses displayed short-term signs of toxicity for the first 45 minutes such as staying at one corner of the cage but were responsive towards provocation and those signs resolved after 45 minutes. Mice that were dosed at 7.5 and 5.5 mg/kg did not show any remarkable clinical signs or abnormal behavior throughout the 14-day period. Therefore, the 50% of the lethal dose (LD50) of this compound was estimated to be 26.25 mg/kg whereas the MTD was estimated to be 17.5 mg/kg for a single dose treatment. Histopathological examination revealed that there were no significant morphological changes in all organs examined except for the 35 mg/kg dose, where the spleen of the mouse showed an increased in multinucleated giant cells, presence of hemosiderin-like pigment (yellow-greenish) and disappearance of clear demarcation between the white and red pulp due to congestion consistent with non-specific reactive changes.

### Subacute toxicity

**Fig 5:**
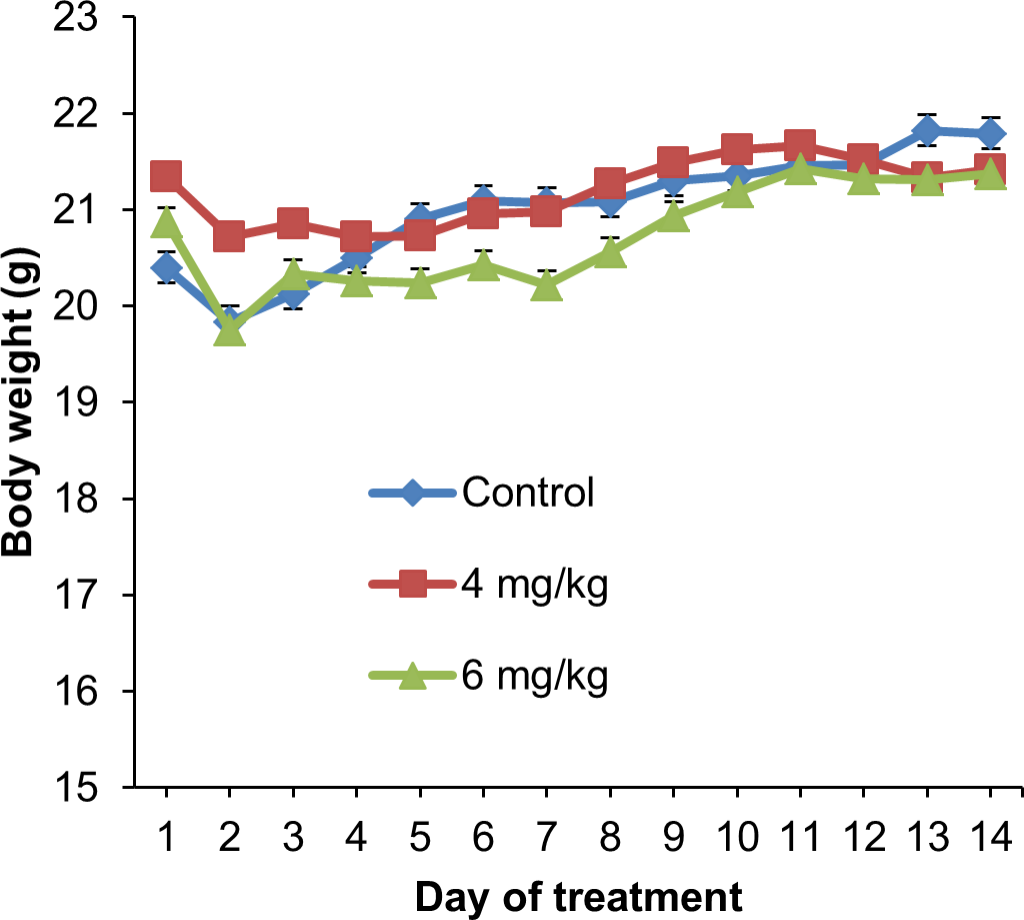
Subacute toxicity study: Mice body weight. Daily monitoring of body weight of non-tumor bearing mice after intraperitoneal administration of normal saline (control group) or Cu(II) complex (4 and 6 mg/kg) daily for 14 days. The values are expressed as mean ± SEM of 3 animals per group.

**Fig 6:**
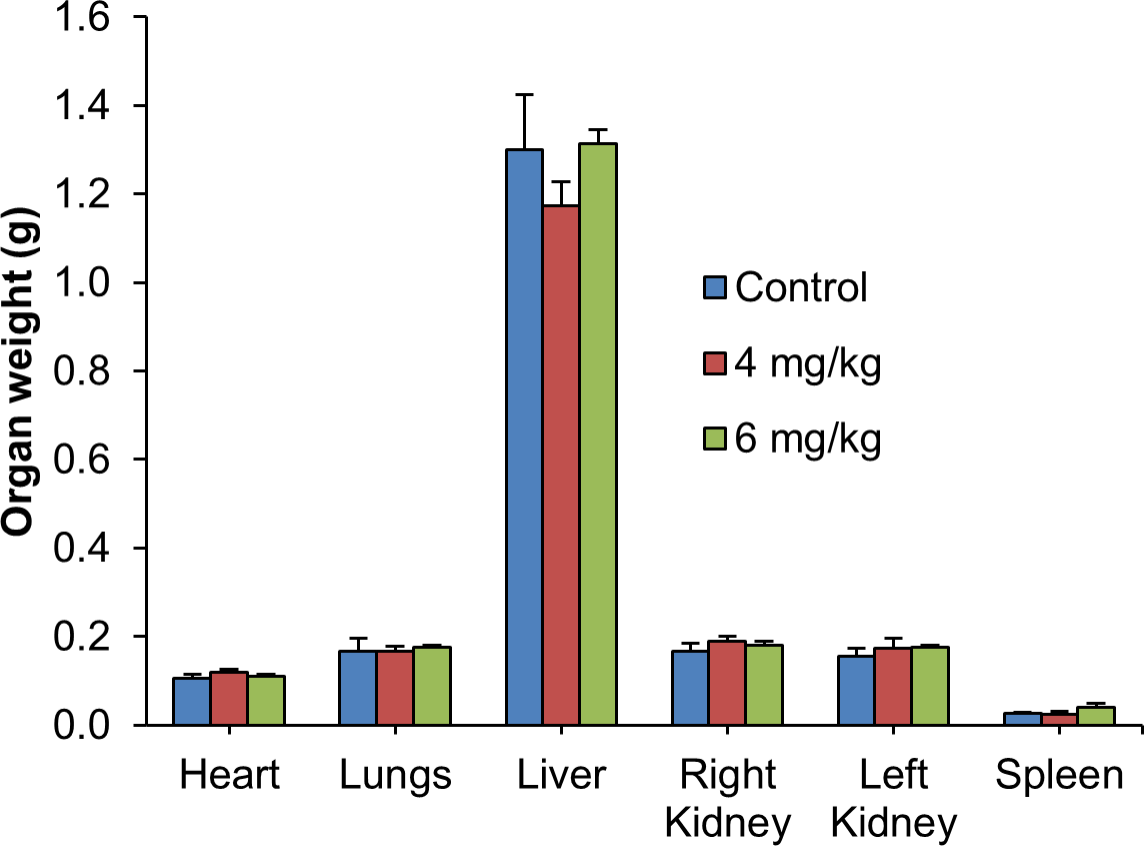
Subacute toxicity study: Organ weight. Weight of organs of non-tumor bearing mice after intraperitoneal administration of normal saline or Cu(II) complex (4 and 6 mg/kg) daily for 14 days. Organ weight measured at day 28 did not show any significant changes between the control and treatment groups. The values are expressed as mean ± SEM of 3 animals per group.

**Fig 7:**
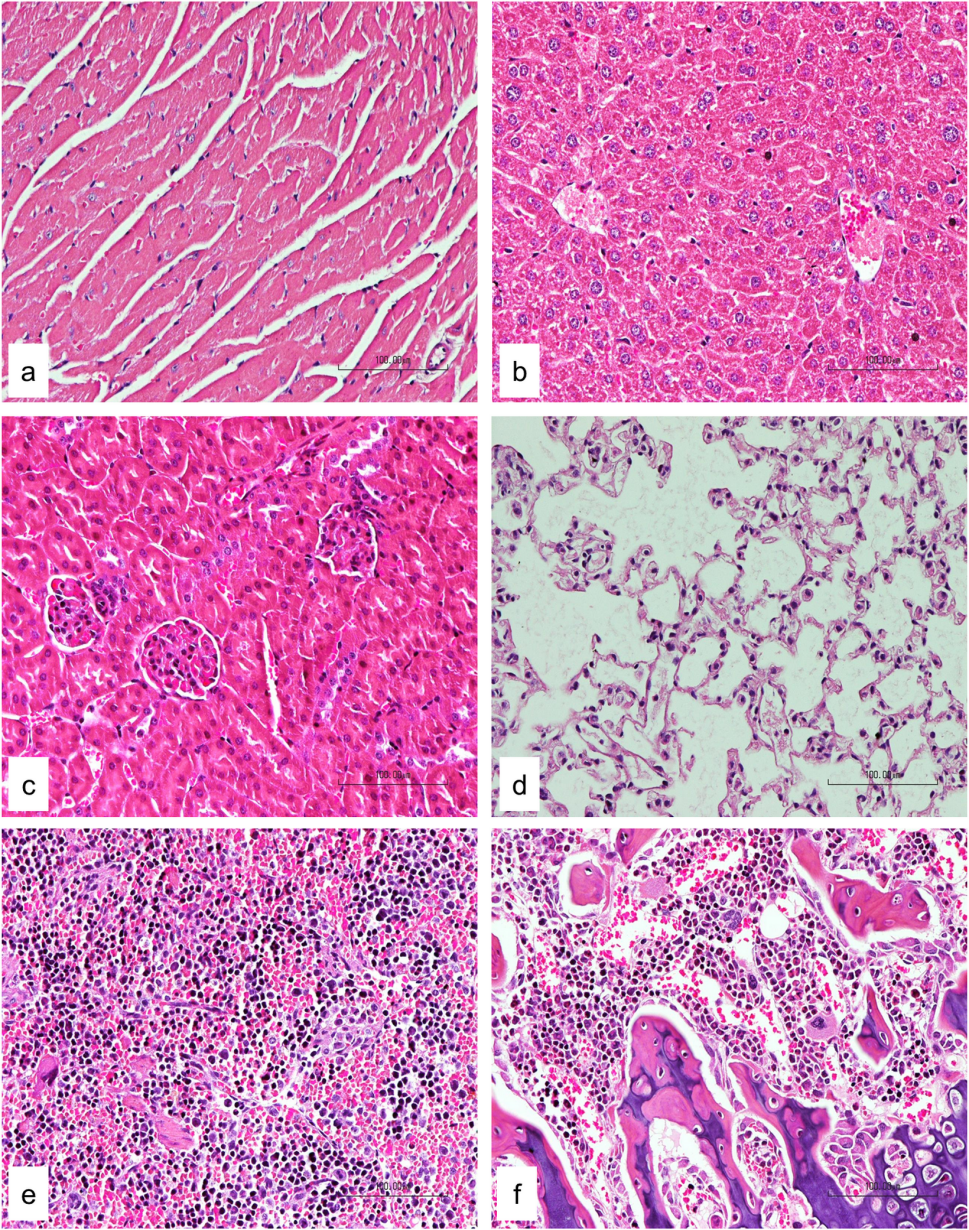
Subacute toxicity study: Histopathology examination of major organs. Histological examination of heart (a), liver (b), kidney (c), lung (d), spleen (e) and femur (f) of 6 mg/kg group did not show any pathological changes (20x magnification).

For subacute toxicity study, three groups of three NSG female mice each were used. Using the formula from NCI, the low dose and high dose were calculated from the MTD value (17.5 mg/kg estimated above) and these were 4 and 6 mg/kg respectively [43]. Mice in the first two groups were administered intraperitoneally with Cu(II) complex at doses of 4 and 6 mg/kg daily respectively for 14 days, while those in the control group received an equal volume of normal saline. The mice were observed for another 14 days to determine the delayed toxicity. No mortality was observed during the course of the study. All mice remained healthy with no obvious differences being noted in the general behavior, physical activities or clinical conditions with respect to the control group. The accumulative intraperitoneal doses administered in these groups were 56.0 and 84.0 mg/kg of the Cu(II) complex, respectively. There was no significant reduction of body weight observed during the treatment period (Fig 5) as well as throughout the whole study. The weight of vital organs was not significantly affected in treatment groups as compared to control group (Fig 6). The site of injections of mice showed some inflammation and induration but these disappeared during the 14-day observation after the last doses were given. Histopathological examination showed no significant morphological changes in all organs in mice treated with normal saline and the test compound (Fig 7). These results indicate that the Cu(II) complex did not cause significant systemic toxicity at the doses tested.

### Hematotoxicity evaluation

Hematotoxicity evaluation is important in the assessment of new anticancer drugs as this type of toxic side effect has limited the widespread use of cisplatin and other anticancer drugs. Values of hematological parameters of mice bearing C666-1-GFP-Luc2, an Epstein-barr virus (EBV) positive NPC cell line [45] xenografts which were treated with 4 and 5 mg/kg of the Cu(II) complex were compared with those from control groups (Table 3). The administration of the Cu(II) complex did not result in a significant reduction of total red blood cells (RBC), hemoglobin (Hb), packed cell volume (PVC) and thrombocytes. This indicated absence of anemia. However, mild leukocytosis due to increase-segmented neutrophils of marginal significance was observed in the 5 mg/kg group with a slight increase of total white blood cells (WBC) count (Table 3) as compared to the control group.

**Table 3:** Effects of Cu(II) complex on hematogical parameters in antitumor efficacy study on NPC.

	Treatment				
	Unit	Control	4 mg/kg	Control	5 mg/kg
RBC	(x10^12^/L)	8.7 ± 0.2	7.8 ± 0.7	8.2 ± 0.3	7.8 ± 0.4
Hemoglobin	(g/L)	144.7 ± 2.9	126.8 ± 10.0	135.7 ± 4.3	133.7 ± 4.4
PVC	(L/L)	0.5 ± 0.0	0.4 ± 0.0	0.4 ± 0.0	0.4 ± 0.0
MCV	(fL)	53.0 ± 0.7	52.9 ± 1.2	52.9 ± 0.9	56.2 ± 1.8
MCHC	(g/L)	314.6 ± 4.5	308.5 ± 4.9	311.6 ± 4.1	308.3 ± 5.0
WBC	(x10^9^/L)	2.2 ± 0.4	2.5 ± 0.5	2.3 ± 0.2	2.9 ± 0.3*
Band neutrophil	(x10^9^/L)	0.1 ± 0.0	0.1 ± 0.0	0.1 ± 0.0	0.1 ± 0.0
Segmented neutrophil	(x10^9^/L)	1.7 ± 0.3	2.0 ± 0.4	1.8 ± 0.2	2.5 ± 0.2*
Lymphocytes	(x10^9^/L)	0.1 ± 0.0	0.2 ± 0.0	0.1 ± 0.0	0.1 ± 0.0
Monocytes	(x10^9^/L)	0.3 ± 0.1	0.2 ± 0.1	0.2 ± 0.0	0.2 ± 0.0
Eosinophils	(x10^9^/L)	0.0 ± 0.0	0.0 ± 0.0	0.0 ± 0.0	0.0 ± 0.0
Basophils	(x10^9^/L)	0.0 ± 0.0	0.0 ± 0.0	0.0 ± 0.0	0.0 ± 0.0
Thrombocytes	(x10^9^/L)	1353.7 ± 45.2	1659.0 ± 120.4	1506.3 ± 63.3	1288.2 ± 155.8
Plasma protein	(g/L)	65.3 ± 2.7	59.5 ± 2.2	62.4 ± 2.6	55.7 ± 2.5

The values were from the efficacy study on mice bearing C666-1-GFP-Luc2 xenograft. Data are expressed as mean ± SEM (group 4 mg/kg Cu(II) complex, n = 3–4; group 5 mg/kg, n = 6). Data for group 6 mg/kg Cu(II) complex was excluded due to insufficient sample size number of animals where only one out of four mice, had survived in the Cu(II) complex treated group.

*p<0.05 was considered significant using t-test. Asterisks denote significant difference compared to control.

Abbreviations: MCV, mean corpuscular volume; MCHC, mean corpuscular Hb concentration.

**Fig 8:**
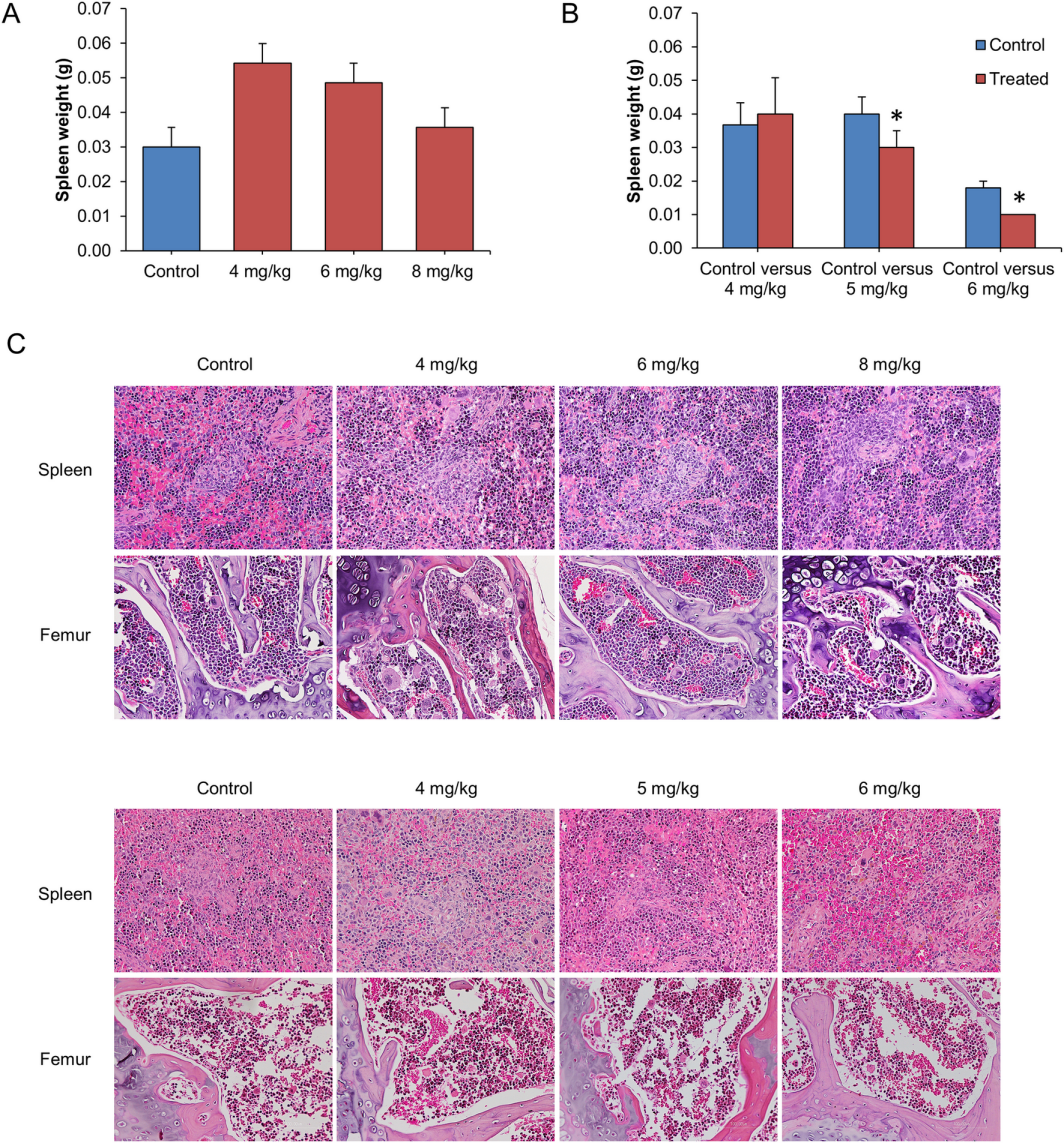
Toxicity study on hematopoietic and lymphoid systems in mice. Hematotoxicity dose response by Cu(II) complex was evaluated by examining spleens and bone marrows (femurs) of mice from the efficacy studies. Weight of spleens from HK1 mice (A) and C666-1-GFP-Luc2 mice (B) treated with different doses of Cu(II) complex. No significant difference was observed in spleen weight except in mice with C666-1 (5 and 6 mg/kg group). (C) Histological examination of spleens and femurs (upper panel, HK1 mice; lower panel, C666-1 mice) did not show any significant pathological changes in all the treatment groups (20x magnification). The values are expressed as mean ± SEM of 3–6 animals per group. *p<0.05 was considered as statistical significant.

We further investigated the effects of Cu(II) complex on spleen and bone marrow (from femur) responses. Spleens from HK1 (a nasopharyngeal squamous carcinoma cell line) [46] bearing mice treated with Cu(II) complex (4, 6 and 8 mg/kg) did not show weight loss (Fig 8A) while those from C666-1-GFP-Luc2 bearing mice treated with 5 and 6 mg/kg groups were observed to have a significantly lower weight when compared with the control groups (Fig 8B). Histopathological analysis of spleen for both groups showed no significant pathological changes (Fig 8C). There was also no evidence of bone marrow suppression, a possible serious side effect of chemotherapy, found in all treated groups. In conclusion, taken together, the Cu(II) complex did not cause significant hematotoxicity in the 4 and 5 mg/kg regimes.

### *In vivo* efficacy of Cu(II) complex in NPC xenograft models

**Fig 9:**
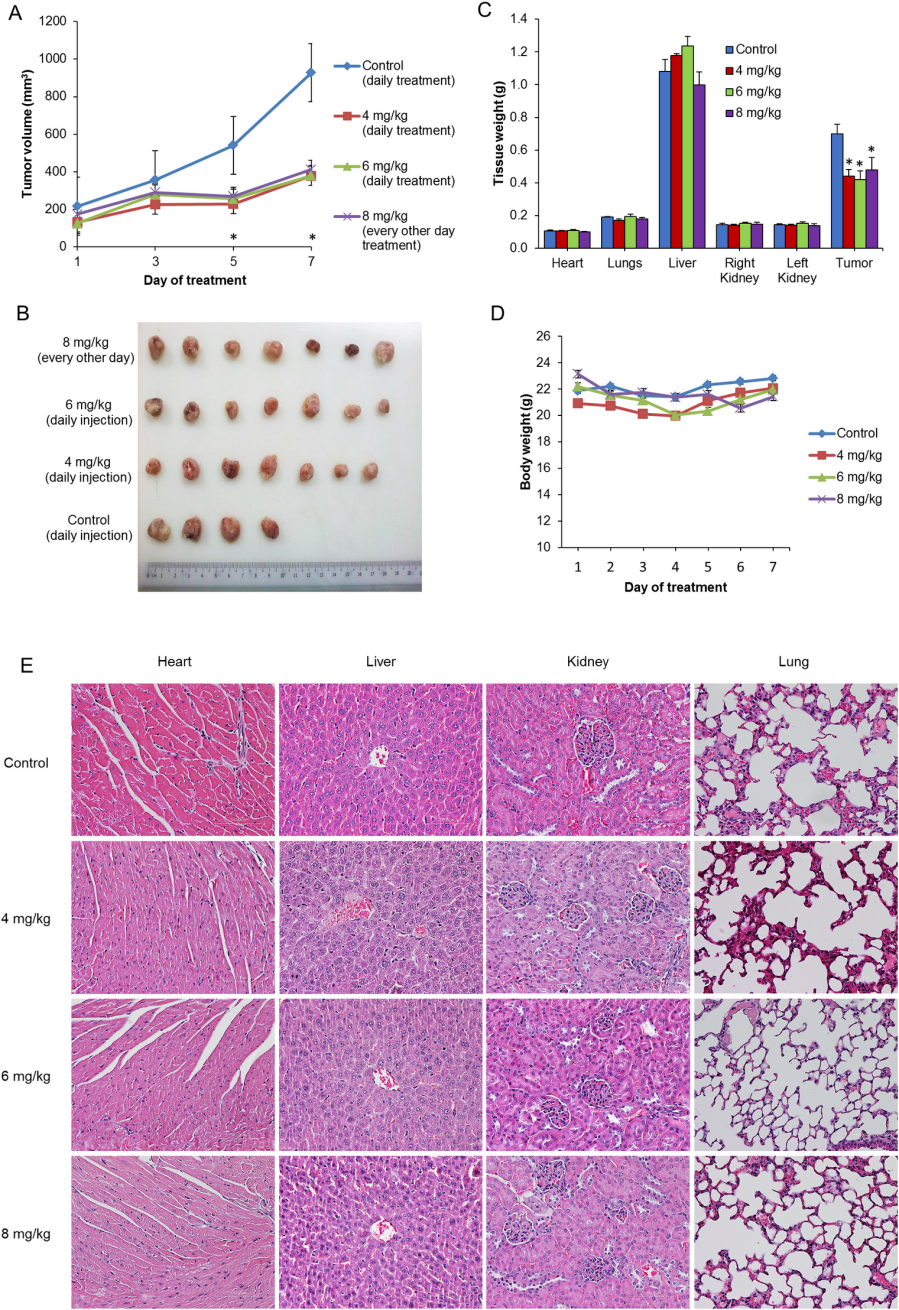
*In vivo* efficacy study on HKI xenograft mice treated with Cu(II) complex. Treatments were given via intraperitoneal injections and the regimes were as described in the Materials and Methods. (A) Tumor volume, (B) excised tumors (C) tissue weight and (D) mice body weight were compared between control and treatment groups. Treatment with Cu(II) complex was found to suppress / inhibit tumor growth and reduce tumor weight. No significant changes were observed in body weight and vital organs. (E) Histological examination of heart, liver, kidney and lung also did not show any significant pathological changes in all the groups (20x magnification). Values are expressed as mean ± SEM of 4–7 animals per group. *p<0.05 compared to control.

In our previous *in vitro* studies, we found that the current Cu(II) complex and other similar ternary Cu(II) complexes containing methylated glycine distinctively exerted greater antiproliferative activity towards and induced more apoptotic cell death in metastatic and cisplatin-resistant breast cancer cells (MDA-MB-231) than in non-tumorigenic (“normal”) breast epithelial cells (MCF10A) [28]. The Cu(II) complex was also found to be effective against cervical (HeLa), ovarian (SKOV3), lung (A549 and PC9), breast (MCF7), lymphoma (Namalwa), leukemia (HL60), and colorectal carcinoma (SW480, SW48, and HCT118) as well as NPC (HONE1, HK1, and C666-1) cell lines with IC50 values (24 h) in the 1.7–19.0 μM range. Recognizing the relative lack of treatment options for advanced NPC compared to other major cancers in Malaysia and based on our previous finding of the anticancer activity against NPC cells, we proceeded to validate the anticancer properties of the Cu(II) complex *in vivo* using NPC xenograft models. To test the compound *in vivo* in the preclinical models, two pilot studies were carried out using the Cu(II) complex. First, the effects of the Cu(II) complex were investigated on HK1 xenograft bearing mice at three different dosage regimens (4, 6 and 8 mg/kg). As shown in Fig 9A, the Cu(II) complex significantly inhibited the growth of HK1 tumor. The excised tumors of untreated and treated mice are shown in Fig 9B. The 4 and 6 mg/kg dose regimens were well tolerated with no significant body weight loss (Fig 9C). Even though treatment with Cu(II) complex at 8 mg/kg every other day up to 3 doses was effective, 2 out of 7 mice were found deadand the experiment had to be terminated. The weight of vital organs was not significantly affected in all treatment groups as compared to control group (Fig 9D). Histopathological examination of heart, liver, kidney and lung did not show any remarkable changes in all the groups (Fig 9E).

**Fig 10:**
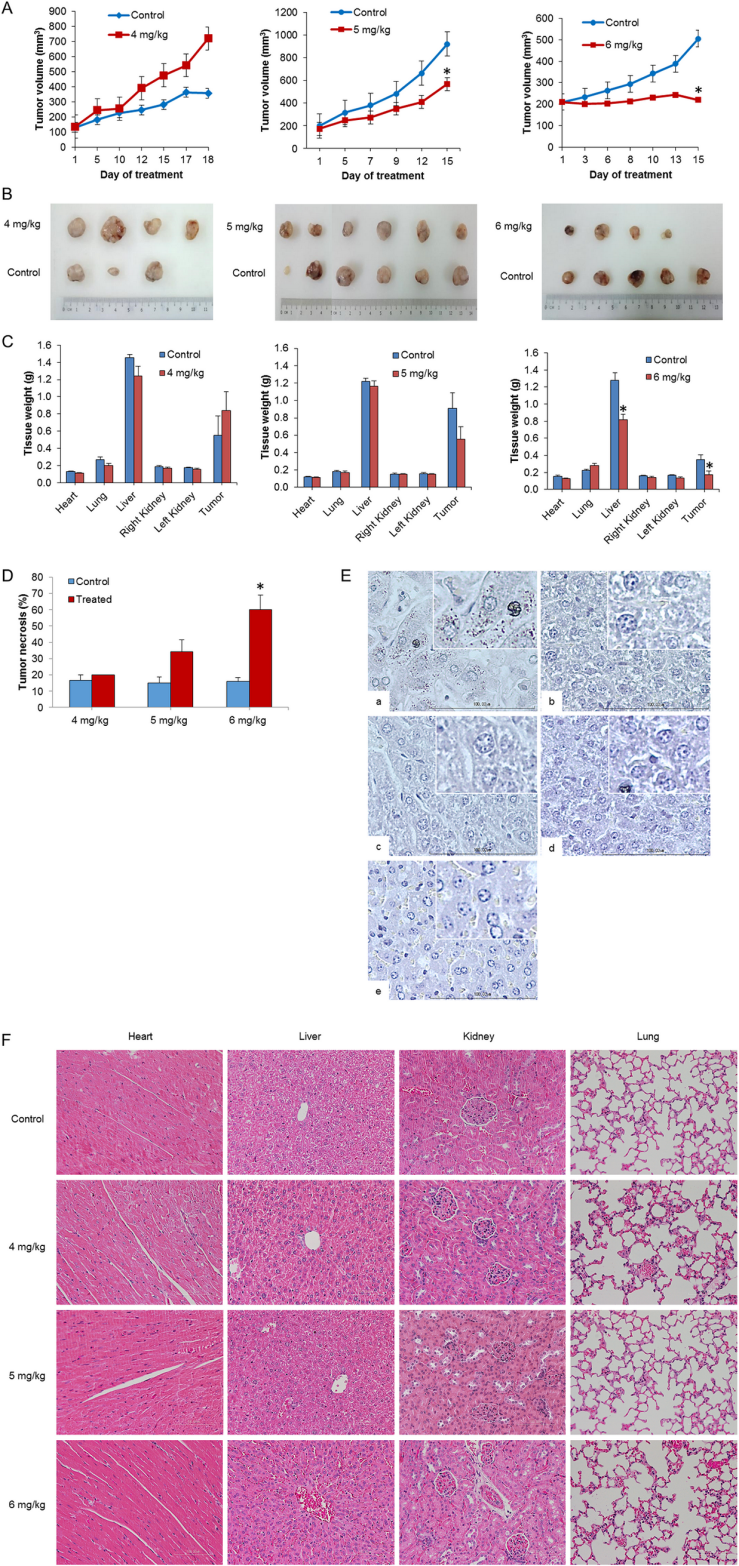
*In vivo* efficacy study on the EBV positive C666-1-GFP-Luc2 xenograft mice treated with Cu(II) complex. (A) Tumor volume, (B) excised tumors and (C) tissue weight were compared between control and treatment groups. Only treatment with Cu(II) complex at 5 and 6 mg/kg were found to suppress/inhibit tumor growth and reduce tumor weight. No changes were observed in the weight of vital organs except for liver in the 6 mg/kg group. (D) Tumor treated with Cu(II) complex exhibited higher tumor necrotic percentage as compared to control groups. (E) Copper staining to stain for the presence of copper deposits in liver. (F) H&E staining of the major organs. Histological examination of heart, liver, kidney and lung did not show any significant pathological changes in all the groups (20x magnification). Values are expressed as mean ± SEM of 3–6 animals per group. *p<0.05 compared to control.

Next, we assessed the anticancer efficacy of the Cu(II) complex on another NPC xenograft, the EBV positive C666-1-GFP-Luc2 cells. As shown in Fig 10A, 4 mg/kg of Cu(II) complex was not effective at suppressing C666-1 tumor even though the treatment was extended to 18 days while 5 and 6 mg/kg treatment groups exhibited a significant tumor growth inhibition as compared to their control groups respectively. The relative sizes of the excised tumors of control mice versus treated mice, at termination of experiment, were consistent with the above results (Fig 10B) The 4 and 5 mg/kg dose regimens were well tolerated with none of experimental mice showing any signs and symptoms of toxicity. On the other hand, even though effective, only one mouse out of four mice in the 6 mg/kg group was well and survived throughout the experiment. Two mice in this group completed the 14 doses but died on the last day of the experiment while one mouse died on day 13 after receiving 12 doses of the Cu(II) complex. The treatment of Cu(II) complex or normal saline had little influence on the body weight of mice (S2 Fig). There were also no significant differences observed in the weight of vital organs except for liver in the 6 mg/kg group (Fig 10C). The Cu(II) complex treatment caused a higher percentage of necrotic area in the tumor xenografts of treated groups compared to control groups, and the percentage necrosis is concentration dependent (Fig 10D). Copper staining of liver sections from mice treated with the Cu(II) complex (4, 5 and 6 mg/kg) showed that all specimens were negative for copper deposits (Fig 10E). Harvested major organs sections stained with hematoxylin and eosin dyes were also examined. Histopathological examination of heart, liver, kidney and lung did not show any obvious abnormal changes in all the groups (Fig 10F). Therefore, no toxicity was found in mice carrying nasopharyngeal C666-1-GFP-Luc2 when treated with Cu(II) complexes at the doses of 4 mg/kg and 5 mg/kg. The underlying cause of death, presumed to be due to toxicity at the dose of 6 mg/kg, could not be ascertained conclusively because although the liver size was smaller in the treated group, the histopathology of the liver appeared normal and no other abnormalities were detected other than lower spleen size.

Growth inhibition of nasopharyngeal HK1 xenograft mice by the Cu(II) complex at 4 mg/kg was effective while that of EBV positive C666-1-GFP-Luc2 xenograft mice required higher doses (5 and 6 mg/kg). Previous work by others showed that a dicopper(II) complex at 4 mg/kg (weekly intraperitoneal injections) was also effective in reducing tumor growth in HepG2 xenograft BALB/c nu/nu nude mice [47]. Cu(II) complex of bis-thiosemicarbazone (GTSC) (5 mg/kg; daily intraperitoneal injection) significantly inhibited tumor growth in human colorectal HTC116 xenograft nude mice [17]. In comparison, a Ruthenium(II) complex, [Ru(dmb)2(salH)]PF6, significantly suppressed BGC823 (gastric cancer cells) xenograft growth *in vivo* at 5 mg/kg and exhibited insignificant nephrotoxicity and nephrotoxicity compared to cisplatin treatment at 4 mg/kg which was slightly less effective but induced considerable damage to liver and kidney [48].

These results suggest that Cu(II) complex displays potentially effective anticancer activity *in vivo* with some toxicity when tested at higher dosages.

## Materials and methods

### Materials and synthesis of Cu(II) complex

Materials and procedure for synthesis of theCu(II) complex was as reported previously [28].

### Cell cultures

The NPC cell lines, HK1 and C666-1-GFP-Luc2 were maintained in an exponential growth phase in RPMI-1640 medium (Gibco, Life Technologies, Carlsbad, CA, USA) supplemented with 10% heat-inactivated fetal calf serum (FCS; Gibco), 100 U/ml penicillin (Gibco), 100 μg/ml streptomycin (Gibco), 0.25 μg/ml fungizone (Gibco) and 1X glutamax (Gibco) at 37°C in a 5% CO_2_ humidified atmosphere. HK1, an EBV-negative NPC cell line previously derived from a patient with a recurrent NPC [46] was kindly provided by Professor George SW Tsao (Hong Kong University, Hong Kong, China). C666-1 is an EBV-positive primary NPC cell line [45]. This C666-1 cell line was later transduced with a lentiviral carrying a green fluorescent protein (GFP) and a luciferase2 gene marker, a kind gift from Dr Marco Herold, Walter and Eliza Hall Institute of Medical Research, Australia [49]. Both cell lines were validated by DNA fingerprinting using AmpFISTR Identifiler Polymerase Chain Reaction (PCR) amplification kit (Applied Biosystems, Foster City, CA, USA) and confirmed free from mycoplasma contamination by using the e-Myco™ Mycoplasma PCR Detection Kit (iNtRON Biotechnology Inc., Seongnam, Korea).

### Experimental animals

Six to eight weeks old, female NOD.Cg-Prkdc^scid^Il2rg^tm1Wjl^/SzJ or also known as NOD scid gamma (NSG, stock no. 005557) (Jackson Laboratory, Bar Harbor, Maine, USA) were used in the acute toxicity, subacute toxicity and *in vivo* efficacy studies. The mice were housed in individually ventilated cages with paper chips bedding (Pure-o’Cel, under specific pathogen-free environment with standard environmental conditions of temperature at 18°C—24°C, with a relative humidity of 45% - 65% and a 12 h dark-light cycle. The mice were allowed free access to acidified drinking water containing co-trimoxazole antibiotics (Xepa-Soul Pattinson Sdn. Bhd., Malacca, Malaysia) and standard pellet diet (Rodent NIH-31M Auto; Altromin Spezialfutter GmbH & Co. KG, Lage, Germany). All experimental protocols were approved by the Animal Care and Use Committee (ACUC) of the Ministry of Health, Malaysia (ACUC/KKM/02[5/2013]). Mice were monitored daily and if they showed any of the signs and symptoms of being unfit or moribund (even before the end point was reached), the mice would be sacrificed immediately.

For the *ex-vivo* experiments using primary hepatocytes, liver cells from Wistar rat were used. The procedures used were approved by the Institutional Animal Ethical Committee (IAEC) of Aurigene Discovery Technologies Limited based on the Committee for the Purpose of Control and Supervision on Experiments on Animals (CPCSEA) guidelines (Aurigene/IAEC/PCD/14 E-11/09-2014). Eight to ten weeks old male Wistar rats (originally from Charles River, USA) with a body weight range of 250–300 g were acclimatized for one-week to standard laboratory conditions. They were fed with standard rat diet (Teklad Global 14% protein diet, UK) and given water ad libitum.

### Rat primary hepatocytes isolation and culturing

Rat primary hepatocytes were freshly isolated using a two-step collagenase method [50] under aseptic condition. Briefly, male rats were anesthetized by intramuscular administration of ketamine (90 mg/kg b.w.) and xylazine (10 mg/kg b.w.). Portal vein was cannulated for perfusion with washing buffer (contains 5 mM KCl, 136 mM NaCl, 25 mM NaHCO_3_, 1.2 mM Na_2_HPO_4_, 6 mM Glucose, 10 mM HEPES-Na, and 0.5 mM EGTA, pH 7.4) at flow rate of 30 ml/min for about 10–15 min until the liver turned into pale yellow when the inferior vena cava was cut for perfusate drainage. Then, the perfusion was switched to collagenase buffer (contains 2.5 mM CaCl_2_ and 0.04% collagenase IV in washing buffer) for about 10 min until the liver tissue became brittle. Carefully, the liver tissue was suspended in the remaining collagenase buffer and mashed gently with sterile blunt spatula to release the hepatocytes. The activity of the collagenase was stopped immediately by adding equal volume of William’s media containing fetal bovine serum (FBS). The cell suspension was filtered through a cell strainer with a 200 μM mesh into sterile 50 ml tubes and centrifuged for 3 min at 25 to 50 x *g* at 4°C (vital hepatocytes have the highest sedimentation rate and will therefore preferentially sediment). The cell pellet was gently resuspended with William’s medium and the suspension was filtered again through a cell strainer with a 100 μM mesh into new centrifugation tubes. The process mentioned above was repeated and the suspension was filtered through a cell strainer with a 60 μM mesh into new centrifugation tubes. After the third centrifugation, the cell pellet was resuspended in 20 ml of William’s media containing fetal bovine serum (FBS) and the viability was checked by trypan blue exclusion method.

### Cytotoxicity in rat primary hepatocytes

12,000 viable rat liver cells in 100 μL growth medium per well were seeded into collagen-coated 96-well plate and incubated overnight at 37°C in a 5% CO_2_ humidified incubator. Prior to addition of compounds, the cells were washed and added with 90 μL of fresh William’s media containing 1% FBS. This was followed by the addition of 10 μL of compound working solution, prepared at 10X of the required concentration in William’s medium, to the respective wells (maximum allowable solvent percentage in this assay was 0.2% DMSO). The plate containing the liver cells was then incubated for 72 h at 37°C in 5% CO_2_ incubator. The liver cells were treated with Cu(II) complex at 100, 50, 25, 12.5, 6.25, 3.125, 1.56, 0.78 and 0.39 μM. In this assay, untreated cells with DMSO alone was used as negative control and cells treated with Tamoxifen as positive control. Four hours prior to the completion of the assay (3-day culture), the media in the wells were removed and the cells in the wells were washed with 1X PBS before finally adding 100 μL fresh PBS to each well. Then, 50 μL of 100 μg/ml resazurin solution was added to each well and the cells were incubated for 2 h at 37°C in CO_2_ incubator. The plate was finally scanned with a fluorescence plate reader (Spectramax Molecular Devices, USA; with excitation of 535 nm and emission wavelength of 590 nm) to determine the viability of the cells in each well.

$$\begin{array}{l} \%\;Viability=\left(Viability\;of\;treated\;cells/Viability\;of\;untreated\;cells\right)\;x\;100\\ \%\;Inhibition=100‑\%\;Viability \end{array}$$

### Cytochrome P450 induction assay

5.0 x 10^5^ viable rat hepatocytes were seeded into collagen-coated 24 well plate in 500 μL William’s medium E (Sigma Aldrich) per well and they were incubated at 37°C in a 5% CO_2_ humidified incubator. After an overnight incubation, the medium in each designated well was replaced with 500 μL of fresh medium containing the specified concentration of Cu(II) complex (1.0, 0.3 and 0.1 μM), dexamethasone (30 μM), 3-methyl cholanthrene (1.0 μM) as reference standards (positive controls) or fresh medium alone (vehicle control). The cells were treated for 3 consecutive days with daily replacement of fresh medium containing compounds. After incubation, the cells were washed with 1X HBSS and the medium was replaced with 300 μL of probe substrates (Midazolam at 10 μM and Phenacetin at 100 μM; prepared in HBSS). The cells were then incubated for a further 20 min at 37°C with mild shaking. The reaction was terminated by adding 600 μL of cold acetonitrile followed by scraping of cells. The above mixture in each well was transferred to a tube, and these tubes were centrifuged at 10,000 rpm for 10 min. The concentration/level of metabolites in the supernatant was monitored by LC-MS/MS.

The extent of fold induction as well as percent of positive control was calculated relative to basal control (untreated) by using the following formula.

%Activity=(averagearearatiooftreated/averagearearatioofbasalcontrol)x100

$$\begin{array}{l} Fold\;induction=\%\;activity/100\\ \%\;of\;positive\;control=fold\;induction\;\left(Cu\left(II\right)\;treated\right)/fold\;induction\;\left(reference\;standard\;treated\right)\;x\;100 \end{array}$$

### CYP450 inhibition assays

Pooled human liver microsomes (Sekisui Xeno Tech, LLC., Kansas City, KS, USA) were incubated with different concentrations of Cu(II) complex or reference standards in buffer containing 0.1% DMSO for 10 min at 37°C and the residual enzyme activity was measured using pooled probe substrates method by pooled LC-MS/MS. Briefly, incubation mixtures were prepared in a total volume of 200 μL with final component concentrations as follows: 0.1 M potassium phosphate buffer (pH 7.4), 1.5 mM NADPH, and 0.25 mg/ml HLM. Probe substrates used were phenacetin (50 μM) for CYP 1A2, bupropion (50 μM) for CYP 2B6, amodiaquine (0.1 μM) for CYP 2C8, tolbutamide (50 μM) for CYP 2C9, S-mephenytoin (120 μM) for CYP 2C19, dextromethorphan (5μM) for CYP 2D6 and midazolam (5 μM) for CYP 3A4. After 20 min of incubation, the reactions were terminated by addition of 200 μL ice-cold acetonitrile (Rankem, Avantor Performance Materials India Limited, Andhra Pradesh, India) containing telmisartan and carbamazepine as internal standards (all probe substrates and standards were from Sigma Aldrich). Each reaction mixture was centrifuged at 3,500 rpm for 5 min and the supernatant was subjected to LC-MS/MS analysis. The percent inhibition was calculated using the following equation:

%Inhibition=100‑[(activityintestwells/activityinbasalcontrolwells)×100]

### NCI60 cell five-dose screen

The Cu(II) complex were submitted to the National Cancer Institute for screening on its panel of 60 cancer cell lines. The protocols used by the NCI have been described previously [51, 52]. Cells of each cell line were seeded in 96-well plates at densities ranging from 5,000–40,000 cells per well depending on the doubling time of individual cell lines. Briefly, cells were exposed to the Cu(II) complex at 0, 0.1, 1.0, 10 and, 100 μM and incubated for 48 h at 37°C in a humidified atmosphere containing 5% CO_2_. The cells were then fixed and stained with sulforhodamine B (SRB) solution to determine their viability, and subsequently calculate the GI50, TGI and LC50.

### Acute toxicity

Determination of acute toxicity was carried out using a method adapted from the NCI [43]. Five mice were randomly separated into five cages. Five doses, i.e. 35.0, 17.5, 12.5, 7.5 and 5.5 mg/kg of body weight of Cu(II) complex were injected intraperitoneally as a single dose into each mouse respectively. After the drug administration, mice were observed hourly for any signs of toxicity, mortality, changes in general behavior and physical activities for the first 4 hours, then 4 hourly for 1 day followed by daily observation for 2 weeks. The signs which were monitored and recorded during this study included hypoactivity, lethargy, hypoapnoea, abnormal gait and posture, tremors, arching and rolling, convulsions, salivation and diarrhea. Mouse death during the period of observation was considered as death due to toxic effects of the test compound. The lethal dose and maximum tolerated dose (MTD) were determined during this study. At the end of the experiment, the survivors were sacrificed for necropsy. Gross and histopathological examinations were performed on the major internal organs including the heart, lung, liver, kidney and spleen.

### Subacute toxicity

Mice were divided into three groups of 3 mice each. The Cu(II) complex was administered intraperitoneally at the doses of 4 and 6 mg/kg daily for 14 days, while the control group received an equal volume of normal saline. The mice were observed for another 14 days to determine the delayed toxicity. Signs of toxicity, mortality, body weight, food consumption and water intake were monitored and recorded daily. Adverse effects such as rapid or consistent body weight loss reaching 20%, blood stained discharges, labored breathing, paralysis, anemia, and other signs and symptoms mentioned in the acute toxicity study, was used as criteria for termination. At the end of the experiment, the mice were sacrificed and the major organs including the heart, lung, liver, kidney and spleen were examined for gross and histopathological changes.

### NPC xenograft models and *in vivo* efficacy assay

2.0 x 10^6^ HK1 or C666-1-GFP-Luc2 cells in 100 μl RPMI media mixed with matrigel (BD Matrigel Basement Membrane Matrix; BD Biosciences, Bedford, MA, USA) was inoculated subcutaneously into the right lower flank of the NSG mice. Tumor growth was monitored and measured every other day using a digital caliper (Mitutoyo America Corporation, Aurora, Illinois, USA) until they reached 100–250 mm^3^. Then, the mice were randomly assigned into treatment groups. The mice bearing HK1 xenograft tumor were divided into 3 treatment groups and a control (normal saline) group. The first batch received normal saline (one injection daily for 5 days), 6 mg/kg (one injection daily for 5 days) and 8 mg/kg (one injection every other day for 3 doses) of Cu(II) complex while the second batch received normal saline (one injection daily for 4 days), 4 mg/kg (one injection daily for 4 days), 6 mg/kg (one injection daily for 4 days) and 8 mg/kg (one injection every other day for 2 doses) of Cu(II) complex *via* intraperitoneal route.

Subsequently, for the efficacy assay using mice bearing C666-1-GFP-Luc2 cells, 3 sets of control group versus experimental group were tested in three independent experiments. Mice were randomly assigned into 4, 5 or 6 mg/kg Cu(II) complex and their control groups respectively. Injections were given once daily for 12 to 18 days. Body weight change, food intake, signs of toxicity/morbidity as well as mortality were observed and recorded daily for the duration of the entire experiment. Tumor volumes were determined using a caliper thrice weekly. Tumor volumes were calculated according to the following formula: Tumor volume (mm^3^) = (length [mm] X width^2^ [mm^2^])/2. At the end point of the studies, mice were placed under anesthesia for blood collection *via* submandibular and cardiac puncture before they were sacrificed for histopathological investigations. Tumors were harvested, imaged, weighed and fixed in formalin.

### Blood analysis

Blood samples were collected into BD Microtainer Tubes containing dipotassium EDTA (Becton, Dickinson and Company, New Jersey, USA) and submitted to the Veterinary Laboratory Services Unit, Faculty of Veterinary Medicine, Universiti Putra Malaysia, Serdang, Malaysia, for full blood count (FBC) analysis.

### Histopathology

Major organs, bones and excised tumors were preserved in 10% neutral buffered formaldehyde solution (Leica Biosystems, Germany) for 24 to 48 h and subsequently processed into formalin-fixed paraffin-embedded tissue blocks following standard tissue processing protocol using Leica ASP300 S (Leica Biosystems, Nussloch, Germany). Fixed bones (skull, femur and tibia) were decalcified with Surgipath Decalcifier I (Leica Biosystems, Germany) for 4 to 6 h depending on size before tissue processing. After paraffin embedding, tissue blocks were sectioned (4 μM thick) using rotary microtome Microm HM340E (Thermo Scientific, Walldorf, Germany) and stained with hematoxylin and eosin (H&E) dyes using Leica Autostainer XL (Leica Biosystems, Nussloch, Germany) before histopathological examination.

### Microscopy

All H&E-stained tissue section slides were examined under Nikon Eclipse Ni microscope (Nikon Instruments Inc., New York, USA) and microscopic images were taken using 20x and 40x objectives. For H&E tumor section slides, the percentage of tumor necrosis was determined qualitatively using microscope by the histopathologist in order to estimate the percentage of necrotic area induced by the Cu(II) complex in the tumors. For determination of femoral bone marrow suppression, low power (10x magnification) objective was used to scan the slides while 100x magnification to assess the degree of cellularity and amount of fat present.

### Copper staining

To demonstrate copper deposits in tissue sections, Copper Stain Kit (Abcam, Cambridge, Massachusetts, USA) containing rhodamine stock solution, acetate buffer solution (pH 8.0) and hematoxylin was used. Firstly, 4 ml of rhodamine stock solution was combined with 46 ml of acetate buffer solution in order to prepare a working rhodamine solution. Tissue sections were prepared, deparaffinized and hydrated in distilled water. Then, a loosely capped staining jar containing working rhodamine was warmed in the microwave oven (Panasonic Corporation, Osaka, Japan). The slide with tissue section was placed in warmed working rhodamine solution and reheated using microwave oven. After that, the jar was capped, carefully agitated using a rotator and allowed to cool to room temperature for 15–20 min. Next, the slides were examined under Nikon Eclipse Ni microscope (Nikon Instruments Inc., New York, USA) for presence of copper deposits and repeated heating and cooling cycle until desired staining intensity was achieved. The slides were rinsed twice with acetate buffer solution for 1 min each, dipped for five times in hematoxylin solution and rinsed thrice with acetate buffer solution. Then the slides were dehydrated in absolute alcohol for 1 min each for three times and cleared in two changes of xylene prior to slide mounting.

### Statistical analysis

Measurements were expressed as mean ± SEM and analyzed using GraphPad Prism 6 (GraphPad Software, San Diego, USA) and Microsoft Excel Version 2013 (Microsoft Corporation, Redmond, USA). A *p*-value of <0.05 between experimental and control groups was considered statistically significant. ANOVA was used to determine differences among different groups over treatment time, followed by post-hoc Tukey’s test. The Student’s t-test was also used for univariate analysis where *p*<0.05 was considered as statistical significance.

## Conclusions

The anticancer efficacy evaluation on the NCI-60 cell lines shows that Cu(II) complex is broad spectrum. The Cu(II) complex did not cause significant induction of both cytochrome P450 (CYP) broad spectrum metabolizing 3A and aromatic hydrocarbon metabolizing 1A enzymes, suggesting it would not cause drug-drug interaction *via* microsomal enzyme induction. However, it moderately inhibited CYP isoforms 1A2, 2C9, 2C19, 2D6, 2B6, 2C8 and 3A4. The Cu(II) complex inhibited the growth of NPC xenografts at doses which did not result in significant toxicity. However, higher doses which resulted in even better inhibition of tumor growth was associated with toxicity. Further work would be useful for the development of these Cu(II) complexes as anticancer agents.

## Supporting information

S1 Fig Results of 5-dose screening of Cu(II) complex on NCI60 cell lines. (TIF)

S2 Fig Body weight of mice bearing C666-1-GFP-Luc2 xenograft during the efficacy study. 4 mg/kg group received 18 days daily treatment while 5 and 6 mg/kg groups received 14 days daily treatment. The values are expressed as mean ± SEM of 3–6 animals per group.
(TIF)

S1 Table Acute toxicity study of mice treated with single dose of Cu(II) complex. (DOCX)
