# Supplementary figures and images for: Ternary copper(II) complex: NCI60 screening, toxicity studies, and evaluation of efficacy in xenograft models of nasopharyngeal carcinoma
Source: PLoS One. 2018 Jan 12;13(1):e0191295. doi: 10.1371/journal.pone.0191295 (PMC5766233; doi:10.1371/journal.pone.0191295)

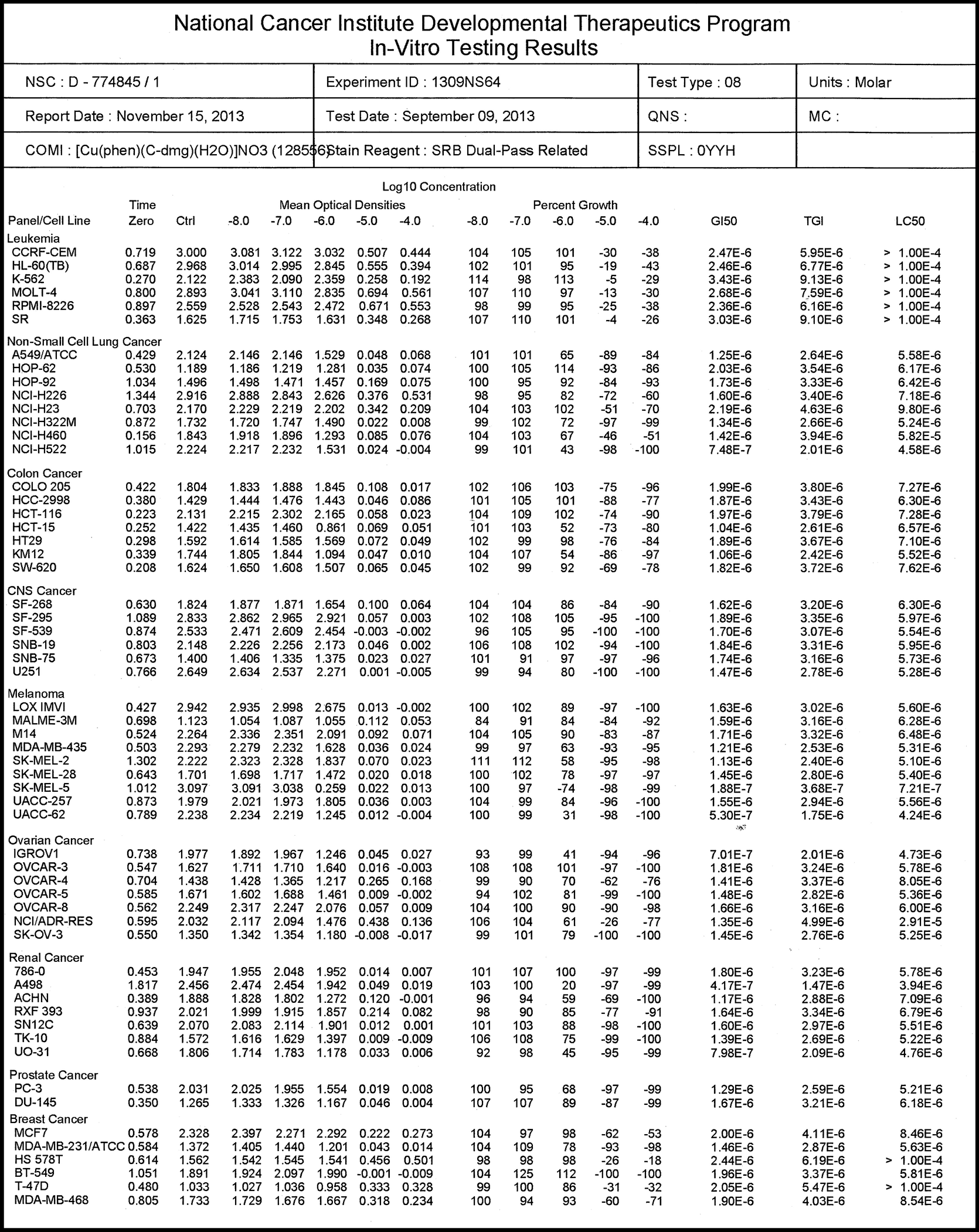

Supplement: S1 Fig — (TIF) [file pone.0191295.s001.tif]

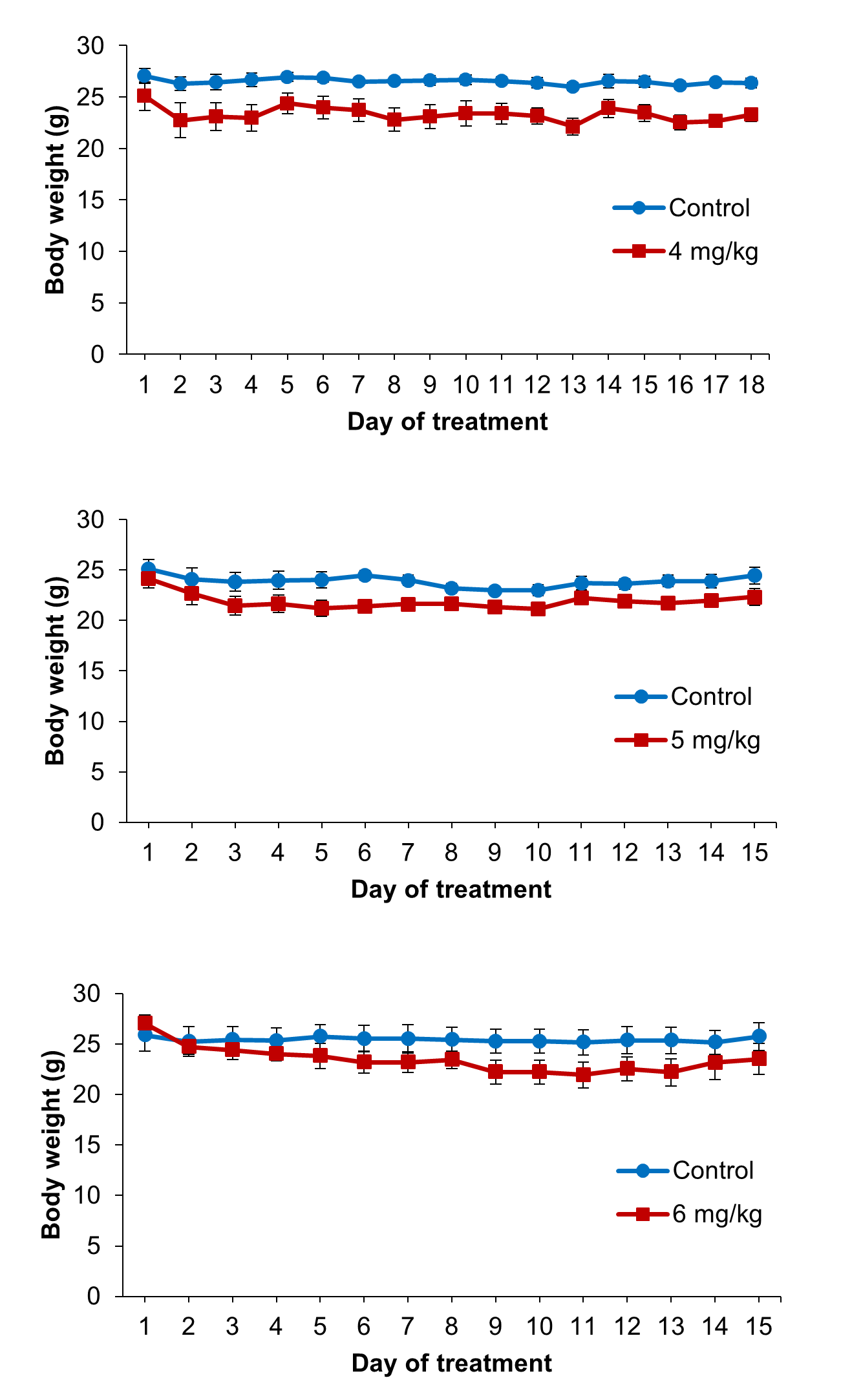

Supplement: S2 Fig — 4 mg/kg group received 18 days daily treatment while 5 and 6 mg/kg groups received 14 days daily treatment. The values are expressed as mean ± SEM of 3–6 animals per group. (TIF) [file pone.0191295.s002.tif]
