# Supplementary material for: Ternary copper(II) complex: NCI60 screening, toxicity studies, and evaluation of efficacy in xenograft models of nasopharyngeal carcinoma
Source: PLoS One. 2018 Jan 12;13(1):e0191295. doi: 10.1371/journal.pone.0191295 (PMC5766233; doi:10.1371/journal.pone.0191295)
Supplement: S1 Table — (DOCX) [file pone.0191295.s003.docx]

**S1 Table.** Acute toxicity study of mice treated with single dose of Cu(II) complex.

|  | | | |
| --- | --- | --- | --- |
| Dosage of Cu(II) complex (mg/Kg) | Observation of mice behavioral and physical changes | | Mortality |
| 35.0 | - Mouse was immobile and staying at one corner of the cage about 30 min after drug injection. - After 0.5, 1, 2, 3 & 4 h, it was still immobile. - Unresponsive to provocation. | It was found dead on Day 2; blood stained urine noted. | Yes |
| 17.5 | - After drug injection, mouse stayed at corner of the cage but resumed its normal behavior and physical activities after 45 min. - Body movement happened. - It showed normal behavior patterns when provoked. | Normal behavior and activities. | No |
| 12.5 | - After drug injection, mouse stayed at corner of its cage but resumed its normal behavior and physical activities after 45 min. - Body movement happened. - It showed normal behavior patterns when provoked. | Normal behavior and activities. | No |
| 7.5 | - No abnormal clinical signs occurred. - It showed normal behavior patterns when provoked. | Normal behavior and activities. | No |
| 5.5 | - No clinical signs occurred. - It showed normal behavior patterns when provoked. | Normal behavior and activities. | No |
